# *Tsc1* haploinsufficiency in Nkx2.1 cells upregulates hippocampal interneuron mTORC1 activity, impairs pyramidal cell synaptic inhibition, and alters contextual fear discrimination and spatial working memory in mice

**DOI:** 10.1186/s13229-020-00340-7

**Published:** 2020-05-06

**Authors:** Nabila Haji, Ilse Riebe, Argel Aguilar-Valles, Julien Artinian, Isabel Laplante, Jean-Claude Lacaille

**Affiliations:** 1 Department of Neurosciences and Groupe de Recherche sur le Système Nerveux Central (GRSNC), Université de Montréal, C.P. 6128, succ. Centre-ville, Montréal, Quebec, H3C 3 J7 Canada; 2 Department of Biochemistry and Goodman Cancer Centre, McGill University, 1160 Pine Avenue West, Montreal, Quebec, H3A 1A3 Canada

**Keywords:** Tuberous sclerosis, Autism mouse model, Contextual fear conditioning, Spatial learning, Inhibitory interneurons, Whole-cell recordings, mTORC1

## Abstract

**Background:**

Mutations in *TSC1* or *TSC2* genes cause tuberous sclerosis complex (TSC), a disorder associated with epilepsy, autism, and intellectual disability. TSC1 and TSC2 are repressors of the mechanistic target of rapamycin complex 1 (mTORC1), a key regulator of protein synthesis. Dysregulation of mTORC1 in TSC mouse models leads to impairments in excitation-inhibition balance, synaptic plasticity, and hippocampus-dependent learning and memory deficits. However, synaptic inhibition arises from multiple types of inhibitory interneurons and how changes in specific interneurons contribute to TSC remains largely unknown. In the present work, we determined the effect of conditional *Tsc1* haploinsufficiency in a specific subgroup of inhibitory cells on hippocampal function in mice.

**Methods:**

We investigated the consequences of conditional heterozygous knockout of *Tsc1* in MGE-derived inhibitory cells by crossing *Nkx2.1*^Cre/wt^;*Tsc1*^f/f^ mice. We examined the changes in mTORC1 activity and synaptic transmission in hippocampal cells, as well as hippocampus-related cognitive tasks.

**Results:**

We detected selective increases in phosphorylation of ribosomal protein S6 in interneurons, indicating cell-specific-upregulated mTORC1 signaling. At the behavioral level, *Nkx2.1*^Cre/wt^;*Tsc1*^f/wt^ mice exhibited intact contextual fear memory, but impaired contextual fear discrimination. They displayed intact spatial learning and reference memory but impairment in spatial working memory. Whole-cell recordings in hippocampal slices of *Nkx2.1*^Cre/wt^;*Tsc1*^f/wt^ mice showed intact basic membrane properties, as well as miniature excitatory and inhibitory synaptic transmission, in pyramidal and Nkx2.1-expressing inhibitory cells. Using optogenetic activation of Nkx2.1 interneurons in slices of *Nkx2.1*^Cre/wt^;*Tsc1*^f/wt^ mice, we found a decrease in synaptic inhibition of pyramidal cells. Chronic, but not acute treatment, with the mTORC1 inhibitor rapamycin reversed the impairment in synaptic inhibition.

**Conclusions:**

Our results indicate that *Tsc1* haploinsufficiency in MGE-derived inhibitory cells upregulates mTORC1 activity in these interneurons, reduces their synaptic inhibition of pyramidal cells, and alters contextual fear discrimination and spatial working memory. Thus, selective dysregulation of mTORC1 function in Nkx2.1-expressing inhibitory cells appears sufficient to impair synaptic inhibition and contributes to cognitive deficits in the *Tsc1* mouse model of TSC.

## Background

Mutations in *TSC1* or *TSC2* genes cause tuberous sclerosis complex (TSC), a disorder associated with epilepsy, autism, and intellectual disability [1, 2]. TSC1 and TSC2 are repressors of the mechanistic target of rapamycin complex 1 (mTORC1), a signaling pathway crucial in the control of neuronal protein synthesis [3, 4]. Thus, alteration in mTORC1-mediated mRNA translation is a cardinal feature of TSC [4, 5]. Given the constellation of symptoms in TSC, molecular dysfunctions in specific brain circuits are likely responsible for these various behavioral changes [5–7].

Consistent with impairments in cognitive function in children with TSC [2], deficiency in hippocampus-dependent cognitive tasks is present in TSC animal models. Mice with heterozygous mutations in *Tsc1* have deficits in hippocampus-dependent contextual fear and spatial learning, in the absence of cerebral pathology [8]. Mice with heterozygous mutations in *Tsc2* have impairments in hippocampus-dependent spatial reference and working memory [9], as well as contextual fear discrimination [9, 10]. These learning and memory deficits are associated with impairments in hippocampal synaptic plasticity. Heterozygous *Tsc2* mice have an abnormally low threshold for induction of late long-term potentiation (LTP) [9], as well as deficits in mGluR long-term depression (LTD) [10]. In the heterozygous Eker rat (*Tsc2*^*+/-*^), both LTP and LTD are impaired [11]. Finally, mGluR LTD is impaired also after postnatal deletion of *Tsc1* in mouse CA1 hippocampus in vivo [12] and in mice with conditional heterozygous *Tsc1* knockout in forebrain excitatory neurons [13].

TSC, as other autism spectrum disorders (ASD), is also associated with an imbalance in excitation/inhibition [6, 14]. Hippocampal circuits are composed of excitatory projection cells and local inhibitory interneurons [15]. Deletion of *Tsc1* in CA1 hippocampal neurons using adeno-associated virus (AAV) delivery of *Cre* recombinase in mice with conditional floxed *Tsc1* (*Tsc1*^*fl/fl*^) enhances excitatory synaptic transmission [12, 16]. Interestingly, with sparse *Cre* expression in a small number of hippocampal neurons, excitatory synaptic transmission is intact but inhibitory synaptic transmission is reduced [6]. Hippocampal inhibitory interneurons are highly heterogenous, and specific cell types are associated with different inhibitory functions [15]. How specific interneurons are affected in TSC to result in impairments of inhibition of principal cells remains largely unknown.

Hippocampal inhibitory interneurons, like their neocortical counterparts, are distinguished by their developmental origin from the medial ganglionic eminence (MGE) or caudal ganglionic eminence (CGE) [15, 17]. Hippocampal MGE-derived interneurons express the homeobox transcription factor Nkx2.1 and include somatostatin (SOM) and parvalbumin (PV) interneurons, as well as nitric oxide synthase (nNOS) expressing ivy and neurogliaform cells [15, 18]. Thus, our goal was to investigate how conditional heterozygous knockout of *Tsc1* in MGE-derived interneurons (*Nkx2.1*^cre/wt^;*Tsc1*^f/wt^ mice) affects hippocampal excitatory and inhibitory cell function, as well as hippocampus-dependent behaviors. We found that *Tsc1* haploinsufficiency in Nkx2.1 cells enhanced mTORC1 activity in hippocampal SOM and PV interneurons. At the behavioral level, *Nkx2.1*^Cre/wt^;*Tsc1*^f/wt^ mice showed impairments in contextual fear discrimination and spatial working memory. At the synaptic level, general miniature excitatory and inhibitory synaptic transmission were intact in pyramidal and Nkx2.1 inhibitory cells of *Nkx2.1*^Cre/wt^;*Tsc1*^f/wt^ mice. However, using optogenetics, we found a decrease in synaptic inhibition of pyramidal cells by Nkx2.1 interneurons, which was rescued by treatment with the mTORC1 inhibitor rapamycin. Taken together, our results establish a link between upregulated mTORC1 signaling in Nkx2.1 interneurons and reduction of their synaptic inhibition of pyramidal cells, and hippocampus-dependent cognitive impairments in mouse, suggesting a possible role of dysregulated mTORC1-mediated translation and synaptic dysfunction in inhibitory cells in TSC.

## Methods

### Animals

Experiments were carried out on mice (8–11-week-old males for behavioral experiments; 6–8-week-old males and females for electrophysiology). Mice were housed 2–5 per cage and given ad libitum access to food and water, in temperature (~ 21 °C) and humidity (~ 55%) controlled rooms with a normal 12-h light/dark cycle. All procedures were approved by the Université de Montréal Animal Care Committee (Comité de déontologie de l’expérimentation sur les animaux, CDEA) and followed the guidelines of the Canadian Council on Animal Care.

### Transgenic mice lines

Conditional floxed *Tsc1* heterozygous knockout mice were generated in MGE-derived interneurons (*Nkx2.1*-Cre mice) by crossing first wild-type males (*Nkx2.1*^wt/wt^) with heterozygous *Nkx2.1*^Cre/wt^ females (The Jackson Laboratory, JAX# 008661) to minimize germline recombination. Then, heterozygous *Nkx2.1*^Cre/wt^ mice were crossed with homozygous *Tsc1*^f/f^ mice (JAX#005680) [19] to generate *Nkx2.1*^cre/wt^;*Tsc1*^f/wt^ or control (*Nkx2.1*^wt/wt^;*Tsc1*^f/wt^) littermates. In experiments with injection of AAV-based constructs (AAV2/9-EF1a-DIO-EYFP [Addgene #27056] and AAV9-EF1a-DIO-hChR2(H134R)-EYFP [Addgene #20298]), heterozygous *Nkx2.1*^Cre/wt^ mice served as the control. Although some germline recombination has been observed by others using Nkx2.1 Cre driver mice [20], we did not observe germline recombination in any of our experiments using EYFP reporters (*n* = 53 *Nkx2.1*^Cre/wt^;*Tsc1*^wt/wt^ mice and 57 *Nkx2.1*^Cre/wt^;*Tsc1*^f/wt^ mice).

### Somatostatin and parvalbumin immunofluorescence

Mice (6–10 weeks old) were deeply anesthetized with sodium pentobarbital (MTC Pharmaceuticals, Cambridge, Ontario, Canada), perfused transcardially first with ice-cold 0.1 M phosphate buffer and then with 4% para-formaldehyde (PFA) in 0.1 M phosphate buffer. The post-fixed brains were cryoprotected in 30% sucrose, and the coronal brain sections (50-μm thick) were obtained using a freezing microtome (Leica SM200R, Germany). Sections were permeabilized with 0.3–0.5% Triton X-100 in 0.1 M phosphate buffer (15 min), and unspecific binding was blocked with 10% normal goat serum in 0.1% Triton X-100 and 0.1 M phosphate buffer (1 h). Sections were incubated overnight at 4 °C with rabbit polyclonal somatostatin 28 antibodies (1/1000; Abcam; Toronto, Ontario, Canada), or at room temperature with mouse monoclonal parvalbumin antibody (1/5000; Millipore; Etobicoke, Ontario, Canada), and subsequently at room temperature with Alexa Fluor® 594-conjugated goat anti-rabbit IgGs (1/500; 90 min; Jackson Immunoresearch Laboratories; West Grove, PA) or Rhodamine-Red™-X-conjugated goat anti-mouse IgG1 (1/200; 90 min; Jackson Immunoresearch Labs). Sections were mounted in ProLong® Gold (Life technologies) and examined using a Nikon microscope (Nikon Eclipse E600; Nikon, Japan) equipped with epifluorescence. Images were acquired with the Simple PCI software (CImaging Systems, Compix Inc., PA).

The number and soma size of somatostatin and parvalbumin-positive cells in CA1 hippocampus were measured in control and transgenic mice. Somatostatin and parvalbumin immunoreactive cell counts and soma size were obtained, in *strata oriens* and *pyramidale*, respectively, from images (typically 4–8 fields of views were analyzed per animal and measures averaged per animal) using Photoshop software. Soma size was measured from the contour of immunofluorescent cells using ImageJ software.

### S6 phosphorylation immunofluorescence

Brain sections obtained as described above were processed for combined parvalbumin or somatostatin and phospho-S6^S235/236^ ribosomal protein immunofluorescence. For parvalbumin, sections were incubated as described above, and subsequently with rabbit polyclonal phospho-S6 antibody (1/400; 48 h; 4 °C; anti-phospho-S6^S235/236^; Cell Signaling, Beverly, MA) and with Alexa Fluor® 594-conjugated goat anti-rabbit IgGs (1/500; 90 min; room temperature). For somatostatin, sections were incubated 48 h at 4 °C with rabbit monoclonal antibody Alexa Fluor® 594-conjugated phospho-S6^S235/236^ antibody (1/100; Cell Signaling) followed by goat anti-rabbit IgG monovalent Fab fragment (1/500; 120 min at room temperature; Jackson ImmunoResearch Labs). Sections were next incubated with rabbit polyclonal somatostatin antibody (1/1000; 24 h, room temperature) followed by FITC-conjugated goat anti-rabbit IgGs (1/500; 90 min; room temperature; Jackson Immunoresearch Labs). Images were acquired using a confocal microscope (LSM510; Carl Zeiss, Oberkochen, Germany) at excitation 488 and 543 nm. Images from parvalbumin or somatostatin immunofluorescence in wild-type and *Tsc1* mice were acquired using the exact same parameters based on wild-type immunofluorescence. Phospho-S6 cell fluorescence was quantified using ImageJ software (National Institute of Health; https://github.com/imagej/imagej1) by comparing integrated density in cells corrected for background fluorescence. Cell fluorescence was measured typically in 24–32 fields of view per animal, and averaged per animal.

### Western blotting

Total hippocampus (10-week-old mice) were collected and protein extracted using ice-cold radioimmunoprecipitation assay buffer containing: 50 mM Tris pH 7.4, 150 mM NaCl, 2 mM EDTA, 1% Triton X-100, 0.5% sodium desoxycholate, 0.1% sodium dodecyl sulfate, 200 μM NaF, 200 μM Na_3_VO_4_, and protease inhibitor (Cocktail inhibitor set I; Calbiochem, Gibbstown, NJ) (20 min, 4 °C). Lysates were centrifuged at 19 000*g* (20 min, 4 °C) and protein concentration from the supernatant was determined according to the bicinchoninic acid method using bovine serum albumin as the standard (Pierce, Rockford, IL). Thirty micrograms of proteins were separated by 12% sodium dodecyl sulfate-polyacrylamide gel electrophoresis and transferred onto polyvinilidene fluoride membrane. The membranes were blocked with 5% non-fat skin milk dissolved in Tris-buffered saline-0.1% Tween 20 pH 7.4 (1 h30, room temperature) and incubated with rabbit polyclonal anti-phospho-S6^S235/236^ (1/1000; Cell Signaling) overnight at 4 °C. Membranes were then incubated with horseradish peroxidase-conjugated anti-rabbit IgGs (1/20000; Jackson ImmunoResearch Labs) for 1 h and 30 min at room temperature. Immunoreactive bands were detected by enhanced chemiluminescence plus (Perkin Elmer, Waltham, MA). Membranes were next stripped with buffer containing 0.2 M glycine pH 2.2, 0.1% sodium dodecyl sulfate and re-probed with antibodies detecting level of total S6 (1/2000; Cell Signaling) and/or tubulin (1/1000; Cell Signaling) overnight at 4 °C. All immunoreactive bands were scanned with a desktop scanner and quantified using Quantity One software (BioRAD, Hercules, CA).

### Electrophysiology

To label Nkx2.1-expressing interneurons, 3–4-week-old *Nkx2.1*^Cre/wt^ and *Nkx2.1*^Cre/wt^;*Tsc1*^fl/wt^ mice were injected in the CA1 hippocampus with AAV2/9-EF1a-DIO-EYFP. Viral particles (0.8 μL; stock solution of at least 10E12 GC/mL; #AV2/9-27056P; Penn vector core; Philadelphia, PA) were injected (0.1 μL/min) at stereotaxic coordinates: (from bregma) antero-posterior − 2.46 mm, lateral ± 1.75 mm, and dorso-ventral − 1.5 mm.

Mice (6–8 weeks old) were anesthetized with isoflurane, and the brain was removed and placed in ice-cold oxygenated (95% O_2_/5% CO_2_) cutting solution containing (in mM) 87 NaCl, 2.5 KCl, 1.25 NaH_2_PO_4_, 7 MgSO_4_, 25 NaHCO_3_, 25 d-glucose, 75 sucrose, 1 ascorbic acid, 3 pyruvic acid, and 0.5 CaCl_2_. Transverse hippocampal slices (300-μm thickness) were prepared using a Vibratome (Leica; VT1000S) and transferred to oxygenated artificial cerebrospinal fluid (ACSF) containing (in mM) 124 NaCl, 5 KCl, 1.25 NaH_2_PO_4_, 2 MgSO_4_, 26 NaHCO_3_, 10 dextrose, and 2 CaCl_2_ (pH = 7.3–7.4; 295–300 mOsmol/L) at room temperature. After a recovery period of at least 45 min, individual slices were transferred to a submersion recording chamber, perfused at 2.5 ml/min with ACSF at 32 °C, and mounted on an upright microscope (Zeiss Axioskop, Toronto, Canada; or Olympus BX50WI, Toronto, Canada) equipped with a long-range water immersion objective (× 40) with Hoffmann (Modulation Optics, Greenvale, NY) or Nomarski optics, epifluorescence, and an infrared CCD camera.

Whole-cell recordings were obtained from CA1 pyramidal neurons or EYFP-positive interneurons under visual guidance using patch pipettes (3–5 MΩ) pulled from borosilicate glass capillaries (World Precision Instruments, Sarasota, USA). Signals were recorded using a Multiclamp 700A amplifier (Molecular Devices) and digitized using Digidata 1440A and pClamp10 software (Molecular Devices). Signals were low-pass filtered at 2 kHz, digitized at 20 kHz, and stored on a PC. Series resistance (15–25 MΩ) was regularly monitored during experiments, and only cells with stable series resistance (changes < 20%) and stable holding current were included.

For current-clamp recordings, the intracellular recording solution contained (in mM) 120 KMeSO_3_, 0.5 EGTA, 10 KCl, 10 HEPES, 4 Mg-ATP, 0.3 GTP-tris, and 10 Di-Na phosphocreatine. Membrane properties were recorded as previously [21] in current clamp at a membrane potential (Vm) of − 60 mV in ACSF containing the NMDA receptor blocker DL-2-amino-5-phosphonovaleric acid (AP5 50 μM; Tocris Biosciences), the non-NMDA receptor blocker 6,7-dinitroquinoxaline-2,3-dione (DNQX 25 μM; Tocris Biosciences), and the GABA_A_ receptor blocker GABAzine (10 μM; Abcam, Canada).

For voltage-clamp recordings of miniature inhibitory postsynaptic currents (mIPSCs) and light-evoked IPSCs in pyramidal cells, the recording solution contained (in mM) 130 CsCl, 10 HEPES, 0.5 EGTA, 2 MgCl_2_, 2 ATP-tris, 0.4 GTP-tris, 5 Di-Na phosphocreatine, and 5 QX-314. Miniature IPSCs were recorded at a Vm of − 60 mV in the presence of the sodium channel blocker tetrodotoxin (TTX 0.5 μM; Alomone Labs), AP5, and DNQX. Light-evoked IPSCs were recorded at a Vm of − 60 mV in the presence of AP5 and DNQX. For voltage-clamp recordings of miniature excitatory postsynaptic currents (mEPSCs) in pyramidal cells, the intracellular solution contained (in mM) 130 CsMeSO_3_, 0.2 EGTA, 8 CsCl, 1 MgCl_2_, 10 HEPES, 3 Mg-ATP, 0.6 GTP-tris, 10 phosphocreatine, and 5 QX-314. Miniature EPSCs were recorded in the presence of TTX and GABAzine.

For EYFP-positive interneurons, voltage-clamp recordings of mEPSCs and mIPSCs were obtained in the same cells using intracellular solution containing (in mM) 130 CsMeSO_3_, 0.2 EGTA, 8 CsCl, 1 MgCl_2_, 10 HEPES, 3 Mg-ATP, 0.6 GTP-tris, 10 phosphocreatine, and 5 QX-314, and ACSF containing TTX and AP5. Miniature EPSCs were recorded at Vm of − 70 mV and mIPSCs at Vm of 0 mV. Miniature E/IPSCs were analyzed using Mini Analysis 6.0.3 software (Synaptosoft Inc., Decatur, GA, USA). For experiments with mEPSCs and mIPSCs recorded in separate cells, the minimum number of miniature events sampled were 200 and 300, respectively, per cell. For experiments with mE/IPSCs recorded in the same cell, a minimum of 220 events was sampled for each. For each cell, mE/IPSC data were binned to avoid oversampling some neurons.

### Optogenetic stimulation

To optogenetically activate Nkx2.1-expressing interneurons with channelrhodopsin (ChR2), 3–4-week-old *Nkx2.1*^Cre/wt^ and *Nkx2.1*^Cre/wt^;*Tsc1*^fl/wt^ mice were injected in the CA1 hippocampus with AAV9-EF1a-DIO-hChR2(H134R)-EYFP (Addgene #20298). Viral particles (0.8 μL; stock solution of at least 10E12 GC/mL; #AV9-20298P; Penn vector core; Philadelphia, PA) were injected (0.1 μL/min) at same coordinates as described above. ChR2 was activated via an optical fiber (1-mm diameter) coupled to a 470-nm custom-made LED system positioned above the slice [22]. The measured LED power was 26 mW at the end of the light guide. To determine input-output function of light-evoked IPSCs, series of light flashes of different duration (0.4–1.8 ms) were given at 30 s intervals and a single cell was recorded per slice.

Given the inherent variability in optogenetic experiments due to differences in viral transduction and ChR2 expression levels, injection site, and virus batch, the following series of precautions were taken to minimize optogenetic response variability. Only slices showing similar strong EYFP expression in CA1 were included. Specificity and efficacy of virus transduction were verified for each animal according to the procedure described for somatostatin and parvalbumin immunofluorescence. Briefly, after recordings, slices were fixed with 4% PFA (4 °C, overnight) and mounted on glass slides. EYFP expression was examined, and images taken, as described for somatostatin and parvalbumin immunofluorescence. In some experiments, slices were re-sectioned (50 μm) for better resolution. For each series of experiments (genotype control vs mutant; acute rapamycin; chronic rapamycin), animal virus injection and slice experiments were always interleaved in control and mutant animals, as well as vehicle or drug treatment, with the experimenter blind to genotype and drug treatment until after data analysis. The same batch of AAV particles was used for a given series of experiments, except for the acute rapamycin experiments (two batches of AAV).

### Rapamycin treatment

The stock solution of rapamycin (50 mg/ml in DMSO; LC Laboratories; Woburn, MA) was prepared and stored at − 20 °C. For rapamycin bath applications, slices were incubated in ACSF containing rapamycin (200 nM) or vehicle (DMSO) at least 30 min prior to recording, as well as during the recordings. For rapamycin chronic administration, 2 days after AAV-ChR2-EYFP injection, mice received an intraperitoneal injection (50 μl) of rapamycin (5 mg/kg) or vehicle (100% DMSO; 2 ml/kg) for 5 consecutive days. Solutions were prepared immediately before injection. Slices were prepared 24 h after the last injection.

### Behavior

Mice were handled daily for 3 days prior to behavioral testing. Behavioral experiments were performed between 9:00 am–3:00 pm. The experimenter was blind to the genotype during testing and analysis.

### Open-field test

For experiments with animal tracking, mice were video-tracked at 25 frames per second and their movements analyzed using a video-tracking system (Smart 3.0, PanLab, Harvard Apparatus), as previously [21]. Before experiments, the animal/image background contrast detection threshold was calibrated by visual inspection.

Open-field tests were conducted as previously [21]. Each mouse was allowed to freely explore a circular open field (60 cm diameter, 25 cm height) for 5 min. Data were analyzed using a custom-made zone pattern (Smart 3.0, Panlab) consisting of three concentric circles (20, 40, and 60 cm diameter, respectively) defining central, intermediate, and peripheral regions. Anxiety measure was obtained from the time spent in the center versus the periphery. Locomotor activity was assessed by measuring the total distance traveled.

### Contextual fear conditioning

Contextual fear conditioning was conducted as previously [21]. Mice were trained in conditioning chambers (17.8 cm × 17.8 cm × 30.5 cm) that were housed in sound- and light-isolated cubicles (Coulbourn Instruments, MA). Chambers contained a stainless-steel grid floor, overhead LED lighting and camera, and were supplied with background noise (60 dB) by an air extractor fan. The experimental protocol was based on Ruediger and coworkers [23] with slight modifications. The training context was rectangular with transparent walls and was cleaned with 1% acetic acid before and after each trial. A novel context was designed to assess contextual discrimination. This context had a circular shape, opaque black and reflective walls, and Plexiglas floor and was cleaned with 70% ethanol before and after each trial. This context was considered novel and distinct to the training context. Freezing was defined as the absence of somatic motility, except for respiratory movements, and analyzed using FreezeFrame (Coulbourn Instruments). Once placed in the conditioning chamber, mice were allowed to freely explore for 2.5 min and then received 5 presentations of unconditioned stimuli (1 s foot shock, 0.8 mA, 30 s interval). To test for long-term contextual fear memory, mice were returned to the training context during a test period of 2.5 min at 24 h after conditioning. To test for contextual discrimination after fear conditioning, a within-subject design was used. On the test day, 5 h after the test in the training context, mice were exposed to the novel context and freezing was assessed during 2.5 min. A discrimination ratio was calculated as the amount of freezing in (training context)/(training context + novel context) [24]. A ratio of 1 indicates that mice were able to discriminate the contexts perfectly, and a ratio of 0.5 means that they were unable to discriminate.

### Barnes maze task

The Barnes maze test was used to assess hippocampus-dependent spatial learning, as previously [21]. The experimental protocol was based on Sunyer and coworkers [25] with slight modifications. In the Barnes maze, mice are trained to use spatial clues to find a small dark escape chamber under the platform called the “escape box.” The maze consists of a gray circular platform, 90 cm in diameter with 20 evenly spaced holes at the edges (PanLab, Spain). The platform is elevated 1 m from the ground to prevent animals from jumping off. All but one of the holes are false-bottomed, while one leads to an escape box. The escape box is retained at the same position relative to the room, while the platform is rotated with each trial to discourage the use of the intra-maze odor cues. In addition, the platform, the starting chamber, and the escape box were thoroughly cleaned (Versaclean 10 %) between every single trial to prevent any possible scent trails. The assignment of escape cage location was balanced among experimental groups. Three reinforcements were used to motivate mice to locate and enter the escape box: ceiling bright lights, the open space of the apparatus itself, and an aversive pulsed noise (76 dB) by a buzzer (TM50, Korg). Before the proper training, animals were first acclimated to the maze in a cylindrical black start chamber placed in the center of the maze for 30 s. Then, mice were allowed to explore the maze for 3 min; the buzzer went on after 30 s. If the mouse failed to find the escape box by the end of the 3 min period, it was gently guided to it. The mouse was left in the escape box for 2 min (buzzer off) or gently guided back if it decided to leave within the 2-min period. During this familiarization trial, the shredded paper was placed within the escape box. Following the adaptation period, mice were trained in 4 sessions daily with an inter-trial interval of 15 min for 4 days (blocks). During the training, mice were left 10 s only in the starting chamber, the buzzer went on right after and mice were left 1 min only in the escape box after each trial (no paper inside, buzzer off). Mice were video-tracked and the number of errors, the latency, and the distance traveled before finding the escape box were collected using Smart 3.0 (PanLab). On the sixth day, the animals were exposed to a probe trial in which the escape box was closed. Mice were allowed to explore the maze for 90 s. The number of errors, the latency, and the distance traveled before the first reaching of the target (primary search) were collected. During the total search (90 s), the time spent in the quadrants (target, left, right, and opposite, excluding a 15-cm-diameter central zone) and the number of visits for each hole were collected. To test for reversal learning, mice were trained on the seventh and eighth day for another series of 4 daily sessions with the escape box moved to a new target (180^o^ position from the previous goal) and were exposed on the ninth day to a memory probe trial.

### Statistical analysis

Data were analyzed using OriginPro (OriginLab) software and are expressed as mean ± S.E.M. Two-group comparisons were carried out using Student’s *t* test. Data were tested for normality using Shapiro-Wilk test. For groups with unequal variance, Welch *t* test was used. Multiple comparisons were made using two-way or three-way repeated measures ANOVA with Bonferroni post hoc comparisons. Cumulative probability distributions were tested for significance with Kolmogorov-Smirnov test. Friedman ANOVA and Mann-Whitney tests were used for nonparametric tests. In figures, asterisks denote statistical significance as calculated by the specified statistical tests (*p < 0.05; **p < 0.01; ***p < 0.001; ns indicates not significant).

## Results

### Conditional heterozygous knockout of *Tsc1* causes cell-specific increase in mTORC1 activity

To examine the effects of conditional heterozygous knockout of *Tsc1* in MGE-derived inhibitory cells, we generated a conditional knockout of *Tsc1* in Nkx2.1-expressing inhibitory cells by crossing *Tsc1*^f/f^ mice with *Nkx2.1*^Cre/wt^ mice.

Since TSC1 is a repressor of mTORC1, we first verified that mTORC1 is affected by the conditional *Tsc1* deletion by assessing phosphorylation of ribosomal protein S6^S235/236^ (p-S6), a downstream effector of mTORC1 signaling, in total hippocampal cells or only inhibitory cells. We assayed p-S6 in total hippocampal cells by western blot assays of hippocampal lysates. Since Nkx2.1-expressing interneurons consist of parvalbumin (PV) and somatostatin (SOM) expressing interneurons [18], we assayed p-S6 immunofluorescence in PV and SOM immunopositive inhibitory interneurons.

**Fig. 1:**
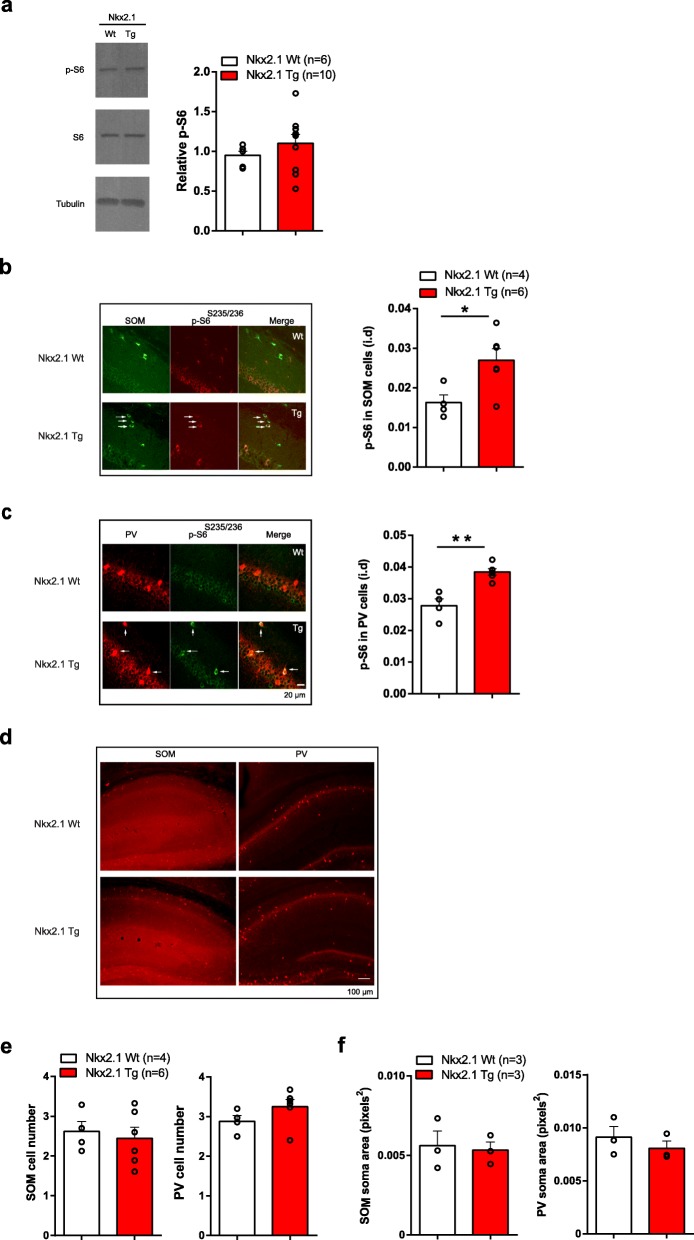
Heterozygous conditional knockout of *Tsc1* in Nkx2.1 interneurons causes cell-specific increase in mTORC1 activity. **a** Representative western blots (left) and summary graph (right) showing unchanged S6 phosphorylation (p-S6 relative to total S6) levels in hippocampal lysates of Tg mice relative to control mice (*n* = 3 Wt (*Nkx2.1*^wt/wt^;*Tsc1*^f/wt^) and 5 Tg (*Nkx2.1*^Cre/wt^;*Tsc1*^f/wt^) mice, analyzed in duplicate; *p* > 0.05, Student’s *t* test). **b**, **c** Representative images (left) and summary graphs (right) showing increased p-S6 immunofluorescence in CA1 SOM (**b**) and PV (**c**) interneurons of Tg mice compared to control mice (*n* = 4 Wt and 6 Tg mice, two independent experiments; *p < 0.05, **p < 0.01, Student’s *t* test). Scale bar in **c** also applies to **b**. **d**–**f** Representative images (**d**) and summary graphs showing unchanged cell numbers (**e**) and soma area (**f**) of CA1 SOM (left) and PV (right) immunopositive interneurons of Tg mice compared to control mice (*n* = 4 Wt and 6 Tg mice for cell number; *n* = 3 Wt and 3 Tg mice for soma area; *p* > 0.05, Student’s *t* tests). Scale bar in **d** applies for all images

In mice with *Tsc1* knockdown in inhibitory cells, mTORC1 signaling was unaffected in total hippocampal cells, as indicated by unchanged hippocampal p-S6 levels in Western blots from *Nkx2.1*^Cre/wt^;*Tsc1*^f/wt^ mutant relative to control *Nkx2.1*^wt/wt^;*Tsc1*^f/wt^ mice (Fig. 1a). However, p-S6 immunofluorescence level was elevated in SOM and PV cells from *Nkx2.1*^Cre/wt^;*Tsc1*^f/wt^ mutants relative to control mice (SOM cells = 269 cells, 142 fields of view in 6 mutant mice versus 206 cells, 105 fields of view in 4 control mice; PV cells = 465 cells, 184 fields of view in 6 mutant mice versus 274 cells, 104 fields of view in 4 control mice; Fig. 1b, c). Thus, mTORC1 activity is selectively upregulated in inhibitory cells, and not in total hippocampal cells, in *Nkx2.1*^Cre/wt^;*Tsc1*^f/wt^ mutant mice.

TSC signaling regulates cell size, morphology, and function [16]. Previous work indicated that conditional homozygous, but not heterozygous, deletion of *Tsc1* in cerebellum resulted in decreases in Purkinje cell number and increases in their soma size of [7]. Therefore, we examined the effect of *Tsc1* heterozygous knockout on inhibitory cell number and soma size. The cell number and soma area of CA1 immunopositive PV and SOM interneurons were unchanged in *Nkx2.1*^Cre/wt^;*Tsc1*^f/wt^ mice relative to control mice (SOM cell numbers = 407 cells, 34 fields of view in 6 mutant mice versus 240 cells, 21 fields of view in 4 control mice; PV cell numbers = 583 cells, 31 fields of view in 6 mutant mice versus 362 cells, 23 fields of view in 4 control mice; SOM cell soma size = 98 cells, 18 fields of view in 3 mutant mice versus 69 cells, 16 fields of view in 3 control mice; PV cell soma size = 44 cells, 14 fields of view in 3 mutant mice versus 51 cells, 14 fields of view in 3 control mice; Fig. 1d–f). Thus, survival and soma size of Nkx2.1-expressing interneurons appear normal in CA1 hippocampus after the conditional heterozygous loss of *Tsc1*.

### Conditional *Tsc1* knockout in inhibitory cells impairs contextual fear memory discrimination

Mice with global heterozygous deletion of *Tsc1* or *Tsc2* show cognitive deficits. *Tsc2*^+/-^ mice exhibit impairments in contextual fear discrimination [26]. *Tsc1*^+/-^ mice display a deficit in contextual fear learning [8]. Thus, we next examined if mice with a conditional heterozygous *Tsc1* knockout in Nkx2.1 inhibitory cells show deficits in contextual fear conditioning and discrimination.

**Fig. 2:**
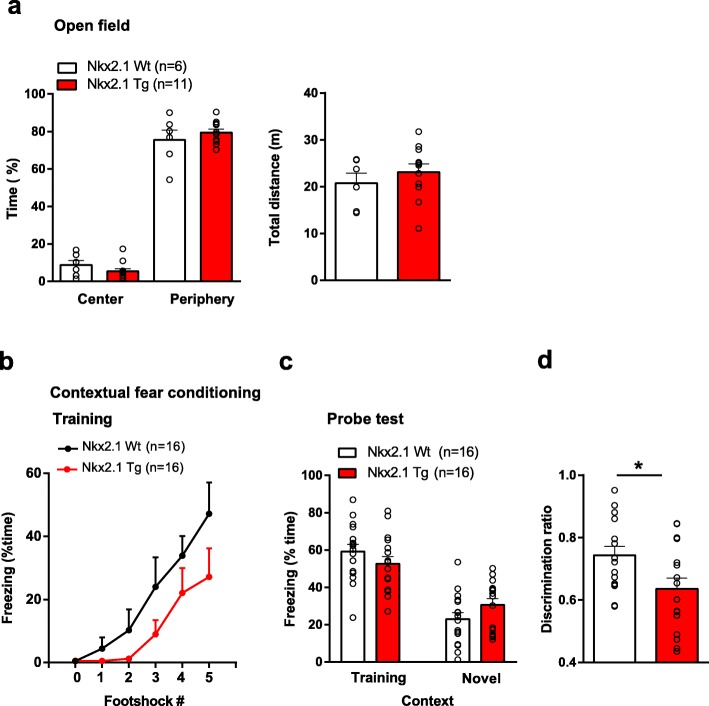
Impairment in contextual fear discrimination. **a** Normal anxiety and locomotion in open-field test. Graphs showing similar (left) time spent in periphery and center of the open field, and (right) total distance traveled by *Nkx2.1*^Cre/wt^;*Tsc1*^f/wt^ mutant and control mice (*n* = 6 Wt (*Nkx2.1*^wt/wt^;*Tsc1*^f/wt^) and 11 Tg (*Nkx2.1*^Cre/wt^;*Tsc1*^f/wt^) mice; ANOVA with Bonferroni post hoc comparisons, *p* > 0.05). **b**–**d** Impaired contextual fear discrimination in *Nkx2.1*^Cre/wt^;*Tsc1*^f/wt^ mutant relative to control mice (*n* = 16 Wt and 16 Tg). Graphs showing similar freezing time during contextual fear conditioning (**b** ANOVA with Bonferroni post hoc comparisons, *p* > 0.05) and in the long-term contextual memory test in the training and novel contexts (**c** ANOVA with Bonferroni post hoc comparisons, *p* > 0.05). Plot of discrimination ratio (freezing time in training context/freezing time in training + novel context) showing impaired discrimination in *Nkx2.1*^Cre/wt^;*Tsc1*^f/wt^ mutant relative to control mice (Student’s *t* test; *p < 0.05)

First, we verified that locomotion and anxiety levels were unaffected in mutant mice using the open-field test (Fig. 2a). *Nkx2.1*^Cre/wt^;*Tsc1*^f/wt^ mutant mice spent more time in the periphery than the center, like control mice. In addition, total distance traveled was similar for *Nkx2.1*^Cre/wt^;*Tsc1*^f/wt^ mutant and control mice. Thus, mice with conditional heterozygous *Tsc1* knockout in Nkx2.1 inhibitory cells display a normal anxiety level and locomotion.

Next, we examined the effects of conditional heterozygous *Tsc1* knockout on contextual fear memory. *Nkx2.1*^Cre/wt^;*Tsc1*^f/wt^ mutant and control mice froze similarly in response to foot shocks during conditioning, indicating normal sensorimotor gating (Fig. 2b). In the long-term memory test, *Nkx2.1*^Cre/wt^;*Tsc1*^f/wt^ mutant and control mice showed similar freezing levels, indicating intact long-term contextual fear memory (Fig. 2c). When exposed to the novel context, *Nkx2.1*^Cre/wt^;*Tsc1*^f/wt^ mutant and control mice showed less freezing than in the training context, but the discrimination ratio was reduced in the *Nkx2.1*^Cre/wt^;*Tsc1*^f/wt^ mutant mice relative to control, indicating an impairment in contextual fear memory discrimination (Fig. 2d). Thus, mice with conditional heterozygous *Tsc1* knockout in inhibitory cells show deficits in contextual memory discrimination.

### Conditional *Tsc1* knockout in inhibitory cells impairs long-term spatial working memory

Mice with global heterozygous deletion of *Tsc1* [8] or *Tsc2* [9] also show hippocampus-dependent spatial learning deficits. Thus, we next examined if mice with a conditional heterozygous *Tsc1* knockout in Nkx2.1 inhibitory cells show deficits in spatial learning.

**Fig. 3:**
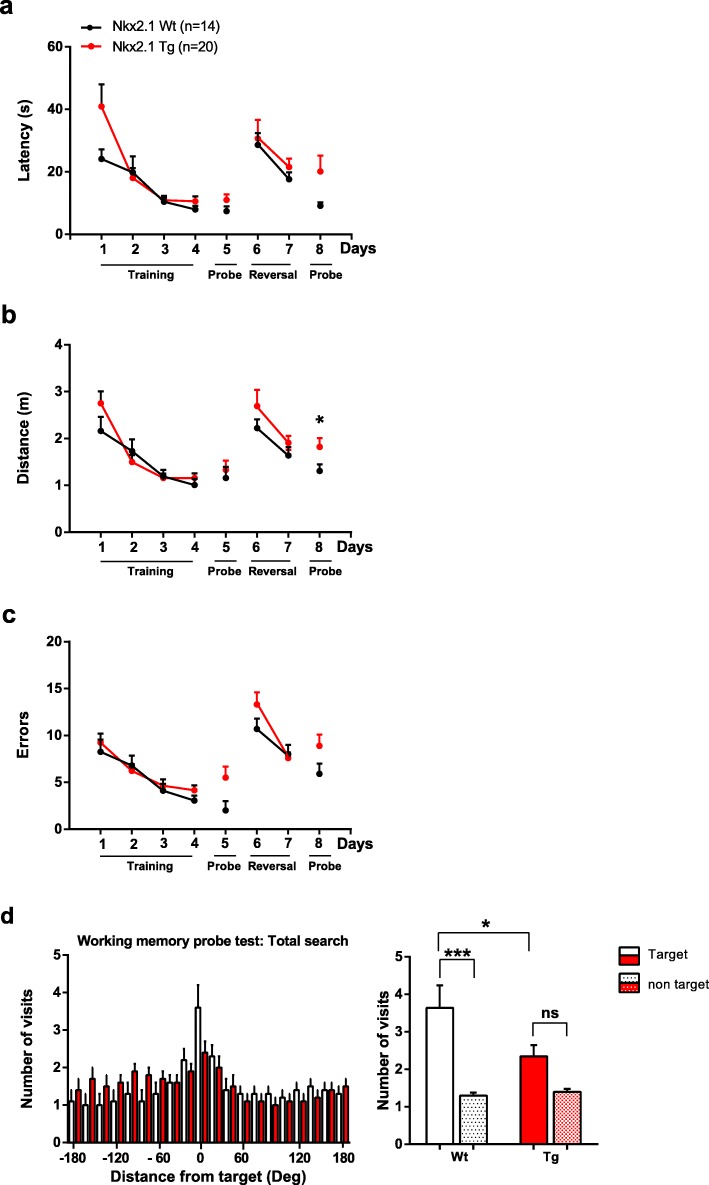
Deficit in spatial working memory in Barnes maze. **a**–**c** Graphs of latency (**a**), distance traveled (**b**), and number of errors (**c**) in reaching the goal box during acquisition (days 1–4), primary search of the long-term *reference* memory probe test (day 5), reversal learning (days 6–7), and primary search of the long-term *working* memory probe test (day 8). *Nkx2.1*^Cre/wt^;*Tsc1*^f/wt^ mutant and control mice performed similarly in all aspects of the tasks (*n* = 14 Wt (*Nkx2.1*^wt/wt^;*Tsc1*^f/wt^) and 20 Tg (*Nkx2.1*^Cre/wt^;*Tsc1*^f/wt^) mice; Friedman ANOVA and Mann-Whitney tests for paired comparisons, *p* > 0.05), except that *Nkx2.1*^Cre/wt^;*Tsc1*^f/wt^ mutant mice traveled a greater distance to reach the goal in the primary search of the working memory probe test relative to control mice (**b**, day 8; Student’s *t* test, *p < 0.05). **d** Impaired precision of long-term memory during the total search of the working memory probe test (day 8). Graphs of number of visits to all escape holes (left) and number of visits expressed as target versus average of all non-target holes (right). Control mice visited the target holes more often than the non-target holes, but *Nkx2.1*^Cre/wt^;*Tsc1*^f/wt^ mutant mice did not (ANOVA with Bonferroni post hoc comparisons, ***p < 0.001, ns *p* > 0.05). In addition, *Nkx2.1*^Cre/wt^;*Tsc1*^f/wt^ mutant mice visited the target holes less often than control mice (*p < 0.05)

We evaluated spatial reference and working memory in *Nkx2.1*^Cre/wt^;*Tsc1*^f/wt^ mice using the Barnes maze, a hippocampus-dependent spatial learning and memory task (Fig. 3). Mice were trained for 4 consecutive days (4 trials per day) and tested on day 5 for long-term reference memory. *Nkx2.1*^Cre/wt^;*Tsc1*^f/wt^ mutant and control mice showed similar decreases in a number of errors, latency, and traveled distance to reach the goal, indicating unimpaired acquisition (Fig. 3a–c). In the memory probe test, *Nkx2.1*^Cre/wt^;*Tsc1*^f/wt^ mutant and control mice displayed a similar number of errors, latency, and traveled distance in the primary search for the target (Fig. 3a–c), indicating unaffected long-term spatial reference memory. Next, mice were trained on days 6 and 7 on a reversal learning task with the new target rotated 180^o^, requiring the mice to learn a new location of the target hole. *Nkx2.1*^Cre/wt^;*Tsc1*^f/wt^ mutant and control mice showed similar decreases in a number of errors, latency, and traveled distance to reach the goal, indicating unimpaired acquisition in the reversal learning task (Fig. 3a–c). On day 8, mice were tested on the working memory probe test. In the primary search, *Nkx2.1*^Cre/wt^;*Tsc1*^f/wt^ mutant mice showed a tendency for increases in a number of errors and latency, and a significant increase in traveled distance to reach the goal relative to control mice (Fig. 3a–c). In addition, during the total search, *Nkx2.1*^Cre/wt^;*Tsc1*^f/wt^ mutant mice showed a reduction in the time spent in the target quadrant relative to control mice, suggesting an impairment in spatial working memory (Fig. 3d). Also, *Nkx2.1*^Cre/wt^;*Tsc1*^f/wt^ mutant mice showed a reduction in the number of visits to the target relative to control mice and did not display a preference for visits to the target vs non-targets, indicating a deficit in spatial memory precision (Fig. 3e–f). Our results suggest that *Nkx2.1*^Cre/wt^;*Tsc1*^f/wt^ mutant mice may have intact long-term spatial reference memory and impaired long-term spatial working memory.

### Conditional *Tsc1* knockout in inhibitory cells impairs synaptic inhibition by Nkx2.1 interneurons

**Fig. 4:**
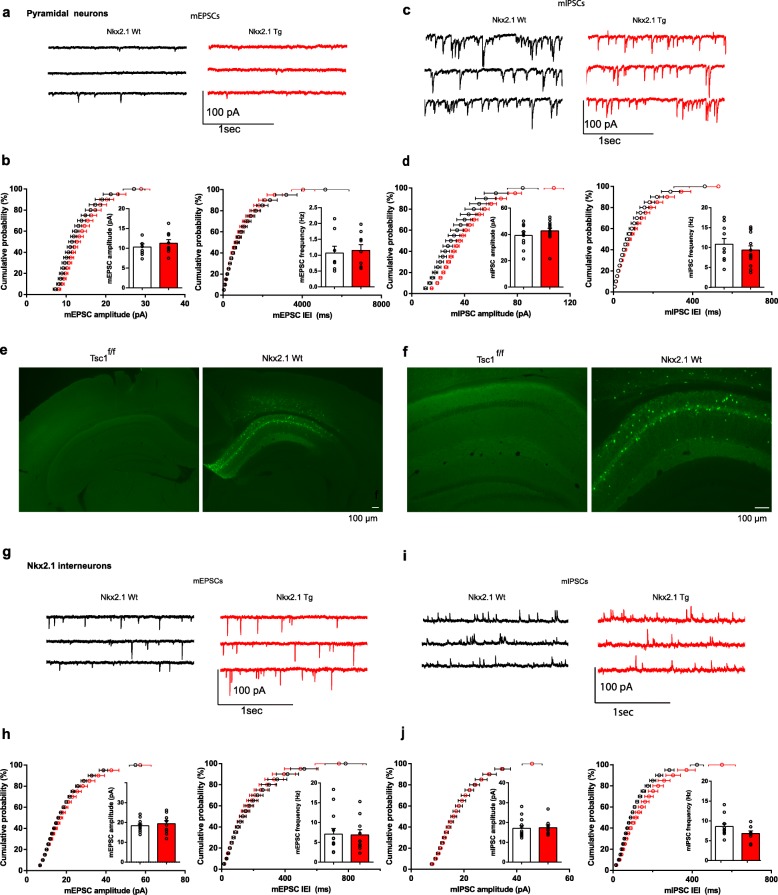
Intact miniature excitatory and inhibitory synaptic transmission in pyramidal cells and Nkx2.1 interneurons. **a**, **b** Representative traces (**a**) and graphs of cumulative probability (**b**) with group data (insets) showing unchanged mEPSC amplitude and frequency (or IEI, inter-event interval) in pyramidal cells of *Nkx2.1*^Cre/wt^;*Tsc1*^f/wt^ mutant relative to control mice (*n* = 8 cells in Wt (*Nkx2.1*^wt/wt^;*Tsc1*^f/wt^) and 9 cells in Tg (*Nkx2.1*^Cre/wt^;*Tsc1*^f/wt^) mice; Kolmogorov-Smirnov tests for cumulative plots, Student’s *t* test for group data, *p* > 0.05). **c**, **d** Representative traces (**c**) and graphs of cumulative probability (**d**) with group data (insets) showing unchanged mIPSC amplitude and frequency (or IEI) in pyramidal cells of *Nkx2.1*^Cre/wt^;*Tsc1*^f/wt^ mutant relative to control mice (*n* = 10 cells in Wt (*Nkx2.1*^wt/wt^;*Tsc1*^f/wt^) and 14 cells in Tg (*Nkx2.1*^Cre/wt^;*Tsc1*^f/wt^) mice; Kolmogorov-Smirnov tests and Student’s *t* tests, *p* > 0.05). **e**, **f** Fluorescence images at low (**e**) and high (**f**) magnification showing the presence (right) of EYFP-positive Nkx2.1 cells in *Nkx2.1*^Cre/wt^ mouse injected with AAV2/9-DIO-EYFP in CA1 hippocampus and the absence of EYFP expression after similar injection in control *Tsc1*^f/f^ mice. Scale bars in images at right apply also to images at left. **g**–**j** Similar electrophysiological data representation showing unchanged mEPSCs (**g**–**h**) and mIPSCs (**i**, **j**) in EYFP-expressing Nkx2.1 interneurons of *Nkx2.1*^Cre/wt^;*Tsc1*^f/wt^ mutant relative to control mice (*n* = 13 cells in Wt (*Nkx2.1*^Cre/wt^;*Tsc1*^wt/wt^) and 9 cells in Tg (*Nkx2.1*^Cre/wt^;*Tsc1*^f/wt^) mice; Kolmogorov-Smirnov tests and Student’s *t* tests, *p* > 0.05). Note that in (**g**–**j**) mEPSCs and mIPSCs were recorded in the same interneurons at − 70 and 0 mV, respectively, resulting in outward mIPSCs (see the “Methods” section)

*Tsc1* knockout by local delivery of Cre in the hippocampus of *Tsc1*^f/f^ mice impairs synaptic inhibition [6] and enhances synaptic excitation [12]. Thus, we next examined the effects of conditional *Tsc1* knockout in inhibitory cells on hippocampal synaptic function. Whole-cell patch-clamp recordings were obtained from CA1 pyramidal cells or from Nkx2.1-expressing interneurons in acute slices from *Nkx2.1*^Cre/wt^;*Tsc1*^f/wt^ mutant and *Nkx2.1*^Cre/wt^ control mice injected with AAV-DIO-EYFP. We confirmed the conditional expression of EYFP by showing the absence of EYFP after injection in control *Tsc1*^f/f^ mice and efficient expression in *Nkx2.1*^Cre/wt^ mice (Fig. 4e–f).

First, we characterized the basic membrane properties of CA1 EYFP-expressing Nkx2.1 interneurons and pyramidal cells. Action potential threshold, half-width and amplitude, as well as resting membrane potential, and afterhyperpolarization amplitude, were unchanged in Nkx2.1 interneurons and pyramidal cells of *Nkx2.1*^Cre/wt^;*Tsc1*^f/wt^ mutant mice relative to control (Table 1), indicating intact general properties of these cells in mutant mice. Although we did not perform extensive cell counts, the number of EYFP-expressing interneurons appeared similar in *Nkx2.1*^Cre/wt^;*Tsc1*^f/wt^ mutant and control mice.

**Table 1:** Membrane properties of Nkx2.1 interneurons and pyramidal cells

Properties	Interneurons		Pyramidal cells	
	Control (*n* = 9)	Tg (*n* = 12)	Control (*n* = 5)	Tg (*n* = 11)
*V*_*m*_ (mV)	− 57.7 ± 1.6	− 60.5 ± 2.2	− 66.5 ± 1.3	− 67.2 ± 0.8
AP threshold (mV)	− 41.6 ± 1	− 41.8 ± 1.5	− 47.2 ± 1.7	− 49.9 ± 0.8
AP half-width (ms)	0.42 ± 0.05	0.33 ± 0.03	0.9 ± 0.1	0.7 ± 0.02
AP amplitude (mv)	67.2 ± 3	67.7 ± 6.2	100.9 ± 4.6	96.3 ± 1.2
AHP amplitude (mV)	− 25.7 ± 1	− 22.1 ± 2.3	− 7.7 ± 1.2	− 9 ± 0.9

*Control Nkx2.1*^Cre/wt^;*Tsc1*^wt/wt^, *Tg Nkx2.1*^Cre/wt^;*Tsc1*^f/wt^, *V*_*m*_ resting membrane potential, *AP* action potential, *AHP* afterhyperpolarization

Next, we determined if conditional *Tsc1* knockout in inhibitory cells affected miniature excitatory and inhibitory synaptic transmission in pyramidal cells. Miniature excitatory postsynaptic currents (mEPSC) amplitude and frequency from CA1 pyramidal cells were similar in *Nkx2.1*^Cre/wt^;*Tsc1*^f/wt^ mutant and control mice (Fig. 4a, b). Similarly, miniature inhibitory postsynaptic currents (mIPSC) amplitude and frequency were unchanged in CA1 pyramidal cells of *Nkx2.1*^Cre/wt^;*Tsc1*^f/wt^ mutant relative to control mice (Fig. 4c, d). These results indicate that conditional deletion of *Tsc1* in Nkx2.1 interneurons does not impair general spontaneous miniature excitatory and inhibitory transmission in pyramidal cells.

Next, we examined if the miniature excitatory and inhibitory synaptic transmission were affected in Nkx2.1 interneurons. Nkx2.1 interneurons are comprised of multiple types of interneurons; thus, we sampled preferentially Nkx2.1 interneurons located at the oriens-alveus border region and in/around stratum pyramidale (approximately 40–60% in each group) to attempt to target equally SOM and PV interneurons. Amplitude and frequency of both mEPSCs and mIPSCs were unchanged in EYFP-expressing Nkx2.1 interneurons from *Nkx2.1*^Cre/wt^;*Tsc1*^f/wt^ mutant relative to control mice (Fig. 4e–j). Thus, general spontaneous miniature excitatory and inhibitory transmission were also unaffected in Nkx2.1 interneurons after conditional deletion of *Tsc1* in these cells.

**Fig. 5:**
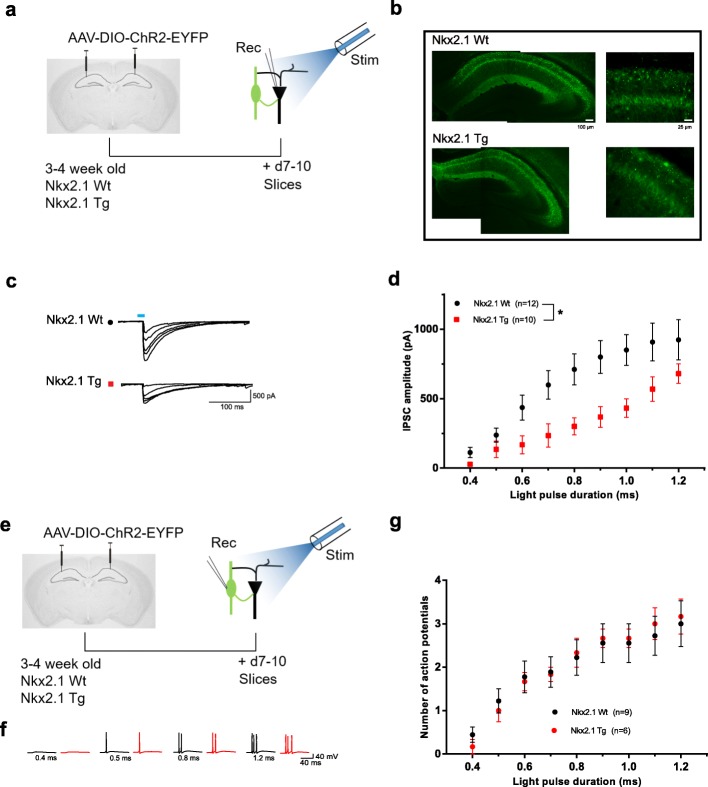
Impairment of synaptic inhibition of pyramidal cells by Nkx2.1 interneurons. **a** Diagram of experimental paradigm with AAV2/9-DIO-ChR2-EYFP injection and pyramidal cell recording in slices from *Nkx2.1*^Cre/wt^;*Tsc1*^f/wt^ mutant and *Nkx2.1*^Cre/wt^ control mice. **b** Representative fluorescence images of EYFP-positive Nkx2.1 cells in CA1 hippocampus showing similar ChR2-EYFP expression in mutant and control mice. Scale bars in top images also applies to bottom images. **c**, **d** Representative traces (**c**) and summary graph (**d**) of input-output function of IPSCs evoked in CA1 pyramidal cells by optogenetic stimulation of Nkx2.1 interneurons in slices from *Nkx2.1*^Cre/wt^;*Tsc1*^f/wt^ mutant (red) and *Nkx2.1*^Cre/wt^ control (black) mice. IPSC amplitude is reduced in mutant compared to control mice (*n* = 12 cells in 6 Wt mice (*Nkx2.1*^Cre/wt^;*Tsc1*^wt/wt^) and 10 cells in 6 Tg mice (*Nkx2.1*^Cre/wt^;*Tsc1*^f/wt^); 2-way ANOVA, *p < 0.05). **e** Diagram of experimental paradigm with AAV2/9-DIO-ChR2-EYFP injection and recordings from EYFP-expressing Nkx2.1 interneurons in CA1 hippocampus. **f**, **g** Representative traces (**f**) and summary graph (**g**) of input-output function of Nkx2.1 interneuron action potential firing evoked by optogenetic stimulation in slices from *Nkx2.1*^Cre/wt^;*Tsc1*^f/wt^ mutant (red) and control (black) mice. Activation of Nkx2.1 interneurons is similar in mutant and control mice (*n* = 9 cells in Wt and 6 cells in Tg mice; 2-way ANOVA, *p* > 0.05)

Next, we sought to determine if conditional deletion of *Tsc1* in Nkx2.1 interneurons could affect specifically their synaptic inhibition of pyramidal cells. To selectively activate inhibition by Nkx2.1 interneurons, we used Cre-dependent expression of ChR2 in hippocampal Nkx2.1 interneurons of *Nkx2.1*^Cre/wt^;*Tsc1*^f/wt^ mutant and *Nkx2.1*^Cre/wt^ control mice, and optogenetic stimulation during whole-cell recordings from pyramidal cells in brain slices (Fig. 5a). Only slices with significant levels of EYFP expression were selected for experiments, and no differences were noted in EYFP expression in *Nkx2.1*^Cre/wt^;*Tsc1*^f/wt^ mutant and control mice (for example, Fig. 5b). Brief pulses of optogenetic stimulation evoked inhibitory postsynaptic currents (IPSCs) in pyramidal cells of both *Nkx2.1*^Cre/wt^;*Tsc1*^f/wt^ mutant and control mice. Small increments in light pulse duration (0.4–1.2 ms) were used to examine the input-output function of optogenetically evoked IPSCs in pyramidal cells (Fig. 5c, d). Input/output functions showed that amplitude of evoked IPSCs was reduced in *Nkx2.1*^Cre/wt^;*Tsc1*^f/wt^ mutant compared to control mice (Fig. 5c, d), indicating an impairment of synaptic inhibition of pyramidal cells by Nkx2.1 interneurons.

To verify that the deficit in synaptic inhibition was not due to an impairment of optogenetic activation of interneurons, we obtained whole-cell recordings from ChR2- and EYFP-expressing Nkx2.1 interneurons and recorded their responses to optogenetic stimulation (Fig. 5e). The input-output functions of evoked firing of Nkx2.1 interneurons were similar in *Nkx2.1*^Cre/wt^;*Tsc1*^f/wt^ mutant and control mice (Fig. 5f, g), indicating similar activation of interneurons by optogenetic stimulation in mutant and control mice.

Together, these results indicate that conditional deletion of *Tsc1* in Nkx2.1 cells does not affect general miniature excitatory and inhibitory synaptic transmission in either pyramidal or Nkx2.1 interneurons, but impairs in a cell-specific manner synaptic inhibition of pyramidal cells by Nkx2.1 interneurons.

### Rapamycin treatment reverses deficits in synaptic inhibition

Impaired synaptic inhibition in hippocampal neurons with *Tsc1* knockout is rescued by mTORC1 inhibition [6]. Thus, we examined whether the deficits in inhibition by Nkx2.1 cells after conditional deletion of *Tsc1* are sensitive to treatment with the mTORC1 inhibitor rapamycin.

**Fig. 6:**
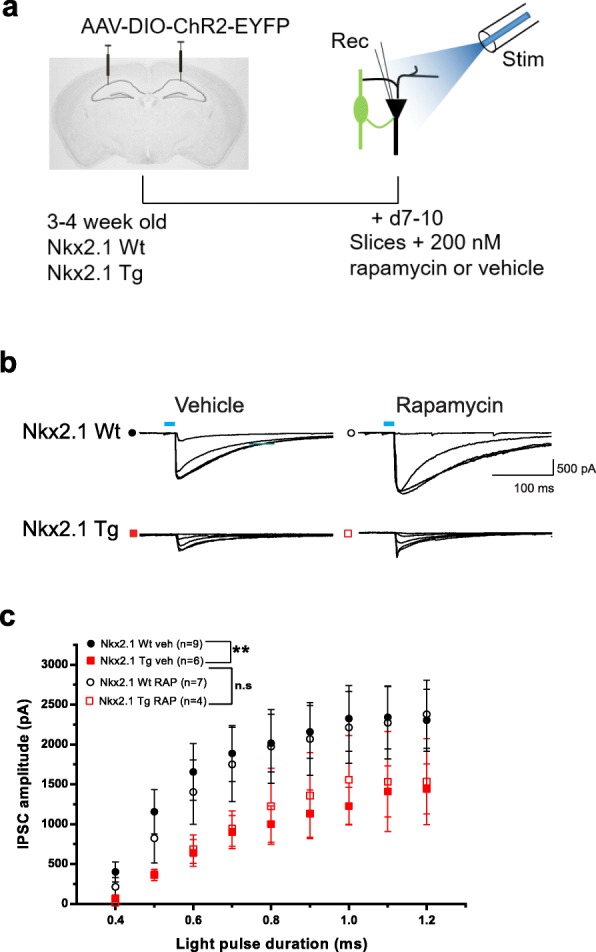
Acute rapamycin treatment did not affect the deficit in synaptic inhibition. **a** Diagram of experimental paradigm for acute treatment of slices with 200 nM rapamycin (or vehicle) and recording of synaptic inhibition of pyramidal cells by Nkx2.1 interneurons from *Nkx2.1*^Cre/wt^;*Tsc1*^f/wt^ mutant and *Nkx2.1*^Cre/wt^ control mice. **b**, **c** Representative traces (**b**) and summary graph (**c**) of input-output function of IPSCs evoked in CA1 pyramidal cells by optogenetic stimulation of Nkx2.1 interneurons in slices from *Nkx2.1*^Cre/wt^;*Tsc1*^f/wt^ mutant (red) and *Nkx2.1*^Cre/wt^ control (black) mice that received vehicle (filled symbols) or rapamycin (open symbols). Acute rapamycin treatment did not affect the reduction of IPSC amplitude in mutant relative to control mice (*n* = 9 cells in 4 animals for vehicle-treated and 7 cells in 4 animals for rapamycin-treated Wt mice (*Nkx2.1*^Cre/wt^;*Tsc1*^wt/wt^); *n* = 6 cells in 4 animals for vehicle-treated and 4 cells in 3 animals for rapamycin-treated Tg mice (*Nkx2.1*^Cre/wt^;*Tsc1*^f/wt^); 3-way ANOVA, Bonferroni tests, **p < 0.01, n.s. not significant)

First, we investigated the effects of acute treatment of slices with rapamycin on synaptic inhibition by Nkx2.1 interneurons after Cre-dependent expression of ChR2 in hippocampal Nkx2.1 interneurons of *Nkx2.1*^Cre/wt^;*Tsc1*^f/wt^ mutant and *Nkx2.1*^Cre/wt^ control mice. Slices were treated with vehicle (DMSO) or 200 nM rapamycin for 30 min prior to and during recordings from pyramidal cells (Fig. 6a). Optogenetic stimulation of Nkx2.1 interneurons evoked IPSCs that were smaller in amplitude in pyramidal cells of vehicle-treated slices of *Nkx2.1*^Cre/wt^;*Tsc1*^f/wt^ mutant compared to control mice (Fig. 6b,c), confirming the deficit of synaptic inhibition in mutant mice. Pyramidal cells that received the acute treatment with rapamycin showed a similar impairment in synaptic inhibition in *Nkx2.1*^Cre/wt^;*Tsc1*^f/wt^ mutant mice compared to control mice (Fig. 6b, c). Thus, acute treatment with rapamycin did not reverse the deficit in synaptic inhibition in mutant mice.

**Fig. 7:**
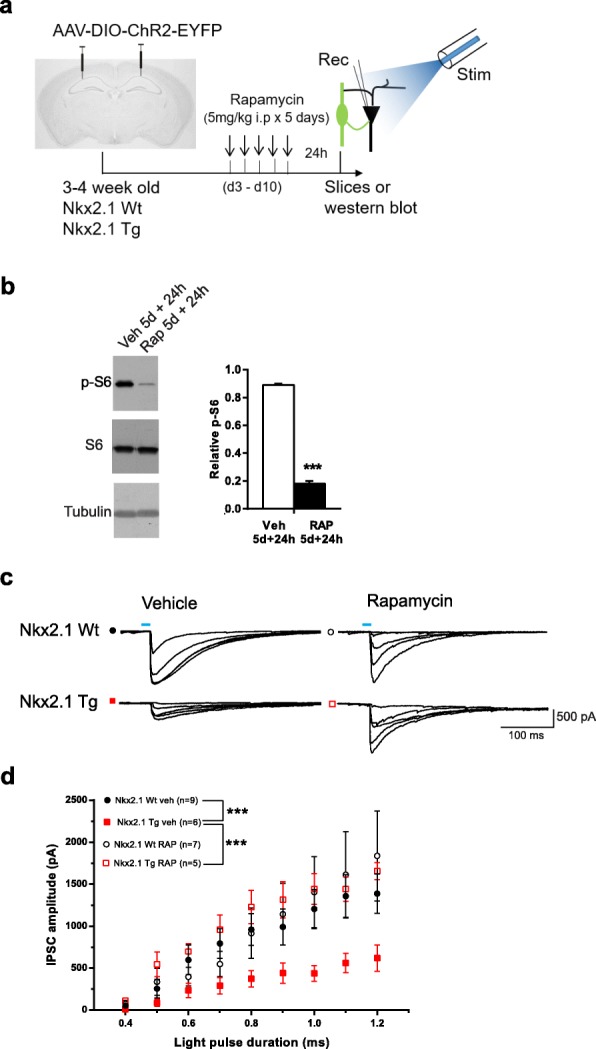
Chronic rapamycin treatment reversed the deficit in synaptic inhibition. **a** Diagram of experimental paradigm of chronic i.p. treatment of *Nkx2.1*^Cre/wt^;*Tsc1*^f/wt^ mutant and *Nkx2.1*^Cre/wt^ control mice with 5 mg/kg rapamycin (or vehicle) and recording of synaptic inhibition of pyramidal cells by Nkx2.1 interneurons. **b** Representative western blots and summary graph showing reduced phosphorylation of ribosomal protein S6 (p-S6; relative to total S6) in hippocampal lysates obtained 24 h after 5-day treatment of control mice with rapamycin or vehicle (*n* = 3 separate experiments in each group; unpaired *t* test, ***p < 0.001). **c**, **d** Representative traces (**c**) and summary graph (**d**) of input-output function of IPSCs evoked in CA1 pyramidal cells in slices from *Nkx2.1*^Cre/wt^;*Tsc1*^f/wt^ mutant (red) and *Nkx2.1*^Cre/wt^ control (black) mice that received vehicle (filled symbols) or rapamycin (open symbols). Chronic treatment with rapamycin reversed the reduction of IPSC amplitude in mutant relative to control mice (*n* = 9 cells in 4 animals for vehicle-treated and 7 cells in 3 animals for rapamycin-treated Wt mice (*Nkx2.1*^Cre/wt^;*Tsc1*^wt/wt^); *n* = 6 cells in 2 animals for vehicle-treated and 5 cells in 4 animals in rapamycin-treated Tg mice (*Nkx2.1*^Cre/wt^;*Tsc1*^f/wt^); 3-way ANOVA, Bonferroni tests, ***p < 0.001)

Next, we examined the effect of chronic treatment of rapamycin. *Nkx2.1*^Cre/wt^;*Tsc1*^f/wt^ mutant and control mice received intraperitoneal injections of 5 mg/kg rapamycin (or vehicle) over 5 consecutive days (Fig. 7a). We verified the efficacy of rapamycin treatment using western blot analysis of S6 phosphorylation, a downstream effector of mTORC1, in wild-type control mice. S6 phosphorylation was reduced in hippocampal lysates from chronic rapamycin-treated mice compared to vehicle-treated mice (Fig. 7b), confirming inhibition of mTORC1 signaling. Next, we examined synaptic inhibition by Nkx2.1 interneurons in slices 24 h after chronic rapamycin treatment of *Nkx2.1*^Cre/wt^;*Tsc1*^f/wt^ mutant and control mice. IPSCs evoked in pyramidal cells by optogenetic stimulation of Nkx2.1 interneurons from rapamycin-treated *Nkx2.1*^Cre/wt^;*Tsc1*^f/wt^ mutant mice were similar to those of control mice and were increased relative to those of mutant mice that received vehicle treatment (Fig. 7c, d). Moreover, rapamycin treatment did not affect IPSCs in control mice compared to vehicle treatment (Fig. 7c, d). Thus, chronic treatment with rapamycin reversed the deficit in synaptic inhibition of pyramidal cells by Nkx2.1 interneurons in mutant mice, without affecting inhibition in control mice, suggesting that impaired inhibition was due to mTORC1 hyperactivation in interneurons of *Nkx2.1*^Cre/wt^;*Tsc1*^f/wt^ mutant mice.

## Discussion

Our results uncover that conditional *Tsc1* haploinsufficiency in a major subgroup of inhibitory cells, MGE-derived interneurons, affects hippocampal function in mice. Conditional heterozygous knockout of *Tsc1* causes an inhibitory cell-specific increase in mTORC1 activity (Fig. 1), an impairment in contextual fear memory discrimination (Fig. 2) and long-term spatial working memory (Fig. 3), as well as a deficit in synaptic inhibition of pyramidal cells by Nkx2.1 interneurons (Fig. 5). Additionally, chronic treatment with the mTORC1 inhibitor rapamycin rescues the deficit in synaptic inhibition (Fig. 7). Thus, our findings establish a link between upregulated mTORC1 signaling in Nkx2.1 interneurons and reduction of their synaptic inhibition of pyramidal cells, and hippocampus-dependent cognitive impairments in mouse, suggesting a possible role of dysregulated mTORC1-mediated translation and synaptic dysfunction in inhibitory cells in TSC.

Our results indicate that in mice with conditional *Tsc1* haploinsufficiency in Nkx2.1 cells, contextual fear learning is intact but contextual fear discrimination is impaired (Fig. 2), and, in addition, spatial learning and reference memory are intact but spatial working memory is deficient (Fig. 3). Interestingly, these changes partially replicate deficits reported in mice with global *Tsc2*^*+/-*^ mutations: impairments in hippocampus-dependent working memory [9] and contextual fear discrimination [9, 10]. Thus, *Tsc1* haploinsufficiency in Nkx2.1 inhibitory cells is sufficient to result in a subset of the hippocampus-related cognitive deficits observed in global *Tsc* mouse models. However, other reported deficits in contextual fear and spatial learning in global *Tsc1*^*+/-*^ mice [8] or in spatial reference memory in global *Tsc2*^*+/-*^ mice [9] were not observed in mice with conditional *Tsc1* haploinsufficiency in Nkx2.1 cells. Thus, these learning impairments likely implicate hippocampal cell types other than Nkx2.1 interneurons.

Our findings shed some light on the mechanisms that might be involved in Nkx2.1 cells to result in the behavioral changes. We found that conditional *Tsc1* haploinsufficiency in Nkx2.1 cells impaired synaptic inhibition of CA1 pyramidal cells by Nkx2.1 interneurons (Fig. 5). Interestingly, the deficit of synaptic inhibition is selective to the inhibition elicited by activation of Nkx2.1 cells, since spontaneous miniature synaptic inhibition of CA1 pyramidal cells and Nkx2.1 interneurons was intact (Fig. 4). These findings are consistent with the notion that spontaneous miniature synaptic inhibition in CA1 pyramidal cells arises from somatic and perisomatic inhibitory synapses from multiple types of interneurons [27–29], whereas optogenetically evoked synaptic inhibition is elicited by selective activation of Nkx2.1 cells. The deficit in synaptic inhibition by Nkx2.1 cells is also unlikely to be due to a loss of Nkx2.1 cells since we found that PV and SOM cell numbers were intact in mice with conditional *Tsc1* haploinsufficiency (Fig. 1). Furthermore, we found that SOM and PV cell soma size was unchanged in mice with conditional *Tsc1* haploinsufficiency (Fig. 1), consistent with the lack of morphological changes in hippocampal principal cells reported in heterozygous *Tsc1*^*+/-*^ mice [8], but in contrast to the hypertrophied hippocampal neurons in mice with homozygous *Tsc1* mutations [30]. Although we did not carry out extensive quantifications, our observations of similar expression of EYFP and EYFP-tagged ChR2 in our whole-cell slice experiments in Wt and Tg mice (Figs. 4 and 5) are consistent with the intact cell numbers in mice with conditional *Tsc1* haploinsufficiency. Finally, the basic membrane and firing properties (Table 1), as well as the excitatory and inhibitory synaptic inputs of Nkx2.1 cells (Fig. 4), were intact in mice with conditional *Tsc1* haploinsufficiency. Thus, the effects of *Tsc1* haploinsufficiency in Nkx2.1 cells appeared to affect most specifically their output synapses and not general cell function. Nkx2.1-expressing cells in the hippocampus consist of multiple interneuron subtypes: SOM and PV interneurons, as well as nitric oxide synthase (nNOS) expressing ivy and neurogliaform cells [15, 18]. Thus, given our findings of deficit of inhibition by Nkx2.1 cells, it will be important in future experiments to determine if the synaptic impairment is present in all, or only in certain, of these identified interneuron subtypes. In addition, the discrepancy between mIPSCs and optogenetic IPSCs might be due to biological reasons such as predominant impairment of mechanisms driving evoked versus spontaneous release, or compensation of synaptic inputs by non-MGE-derived interneuron populations. Nonetheless, our finding of efficient conditional postnatal Cre recombination in Nkx2.1 inhibitory cells (Figs. 4 and 5) provides a great tool for the understanding of Nkx2.1 expression and function of these interneurons in the hippocampus.

In our experiments, the amplitude of optogenetically evoked IPSCs showed variability across different series of experiments (for example, IPSCs in Wt Fig. 5d and Fig. 6c). Due to the inherent variability in optogenetic experiments, we took experimental precautions (see the “Methods” section) to minimize optogenetic response variability within each series of interleaved experiments (i.e., Figs. 5, 6, and 7). In addition, as a control for transfection efficiency during experiments, optogenetically evoked IPSCs were only recorded in slices of Wt and Tg mice with similar efficient EYFP expression verified by fluorescence microscopy (Fig. 5b). Moreover, we verified that optogenetic activation of Nkx2.1 cells was efficient and similar in Wt and Tg mice (Fig. 5f, g). Although the latter may not represent optogenetic activation of Nkx2.1 axon terminals, efficient expression in axon terminals was confirmed by the observation of EYFP-tagged ChR2 in CA1 regions containing Nkx2.1 interneuron axon terminals (*stratum pyramidale* and *stratum lacunosum-moleculare*; Fig. 5b). It should also be noted that differences in light-evoked IPSCs across different series of experiments may be due to other possible extraneous variables arising from different treatment conditions (Fig. 5, Wt and Tg mice comparison with no other treatment; Fig. 6, Wt and Tg mice comparison with acute slice vehicle versus drug treatment; Fig. 7, Wt and Tg mice comparison with i.p. injection of vehicle versus drug). Finally, we observed that the phenotype (reduction of optogenetically evoked IPSC amplitude in Tg relative to Wt mice) was similarly significant in all three series of independent experiments (Fig. 5, Tg vs Wt mice; Fig. 6 vehicle-treated Tg vs Wt mice; Fig. 7 vehicle-injected Tg vs Wt mice). Moreover, the rapamycin-treated group was significantly different from the vehicle only for the series of experiments in Tg mice after i.p. treatment (rescue of IPSC amplitude deficit; Fig. 7). Thus, our findings of significant results within individual series of experiments and that are repeatable across independent series of experiments suggest that although optogenetically evoked IPSC amplitude may show some variability across different series of experiments, these optogenetic responses show consistency within a given series of interleaved experiments allowing to detect significant differences and reach significant conclusions. Nonetheless, it would be of interest in future studies to test for such an inhibitory deficit by Nkx2.1 interneurons using triple transgenic mice with a ChR2^f/wt^ allele for stable ChR2 expression (covering all MGE interneurons and avoiding injection artifacts) or performing paired recordings between EYFP+ interneurons and pyramidal cells in both genotypes.

Our results also indicate that dysregulated mTORC1 signaling in Nkx2.1 is responsible for the impaired inhibition. We found that mTORC1 signaling was elevated in SOM and PV interneurons, the two major subtypes of Nkx2.1 inhibitory interneurons, but was unaffected in total hippocampal cells, in mice with conditional *Tsc1* haploinsufficiency (Fig. 1). This interneuron-specific mTORC1 hyperactivation in Nkx2.1 cells is consistent with the well-documented role of dysregulated mTORC1 in hippocampal principal cells of mice with global heterozygous *Tsc2*^*+/-*^ [9, 10] or with conditional floxed homozygous *Tsc1*^*c/c*^ [6, 9, 30]. Our observation that a 5-day treatment with the mTORC1 inhibitor rapamycin rescued the deficit in synaptic inhibition by Nkx2.1 cells in mice with conditional *Tsc1* haploinsufficiency without affecting inhibition in control mice (Fig. 7) clearly shows that mTORC1 hyperactivation was causing the deficit in synaptic inhibition.

The mechanism by which mTORC1 hyperactivation in Nkx2.1 cells results in reduced inhibition remains to be determined. Since acute inhibition of mTORC1 by bath application of rapamycin in slices failed to rescue the deficit in inhibition (Fig. 6), long-term effects of mTORC1 activation on inhibition may be involved. It is well documented that mTORC1 is a critical regulator of protein synthesis involved in long-term plasticity of excitatory synapses in hippocampal pyramidal cells [31, 32]. However, mTORC1 is also involved in the regulation of synaptic plasticity in hippocampal inhibitory interneurons [33–35] and these mechanisms could be involved in the deficits in inhibition. First, a long-term depression of inhibitory synapses onto pyramidal cells is elicited by repeated theta-burst episodes of the postsynaptic firing of pyramidal cells and this depression is dependent on mTORC1-mediated protein synthesis in presynaptic inhibitory interneurons [35]. If such mTORC1-mediated long-term depression of inhibition takes place at inhibitory synapses made by Nkx2.1 interneurons on pyramidal cells, it could explain that hyperactivation of mTORC1 in Nkx2.1 cells leads to depression of their output synapses by conditional *Tsc1* haploinsufficiency. Second, mTORC1 activation by high-frequency synaptic stimulation results in a long-term potentiation of intrinsic excitability and action potential firing in hippocampal PV cells [34]. If hyperactivation of mTORC1 in Nkx2.1 cells leads to impairment in long-term potentiation of PV cells firing, this may result in a reduction of activation of their output inhibitory synapses onto pyramidal cells. Finally, a long-term mGluR1a-mediated and mTORC1-dependent LTP is present at excitatory synapses onto SOM interneurons [21, 33]. In mice with conditional *Tsc1* haploinsufficiency in SOM interneurons resulting in mTORC1 hyperactivation in SOM cells, the threshold for induction of LTP is lowered, but when elicited using normal induction conditions the LTP is blocked [21]. Similar changes in plasticity of excitatory synapses onto SOM interneurons should be present in mice with *Tsc1* haploinsufficiency in Nkx2.1 cells. Since Nkx2.1 interneurons in the hippocampus include PV and SOM interneurons, as well as nNOS-expressing ivy and neurogliaform cells [15, 18], the reduction in synaptic inhibition in conditional *Tsc1* haploinsufficiencey in Nkx2.1 cells may arise from multiple interneuron-specific mTORC1 mechanisms in these cells.

Interestingly, mTORC1 is implicated in mGluR-mediated protein synthesis-dependent long-term depression (mGluR-LTD) of excitatory synapses in hippocampal pyramidal cells [36, 37] (but see [38]). In mice with pan-neuronal or forebrain principal cell-specific *Tsc1* haploinsufficiency, mTORC1 signaling is hyperactivated and mGluR-LTD is impaired [10, 12, 13]. Given the depression of synaptic inhibition in mice with *Tsc1* haploinsufficiency in Nkx2.1 cells, synaptic deficits resulting from *Tsc1* haploinsufficiency and mTORC1 hyperactivation appear distinct in excitatory and inhibitory cells, further highlighting the multiple cell-specific mechanisms that may occur with *Tsc1* haploinsufficiency. These findings are consistent with the multiple cortical cell type-specific (including excitatory, PV, and SOM cells) molecular changes recently identified by single-cell genomic analysis in ASD [39].

Recently, a deficit in miniature synaptic inhibition was reported in layer 2–3 pyramidal neurons of the visual cortex of mice with pan-neuronal *Tsc1* haploinsufficiency [40]. Mice with conditional *Tsc1* haploinsufficiency in PV or SOM interneurons did not show such reduction in miniature synaptic inhibition, suggesting that the reduction of inhibition in pan-neuronal *Tsc1* may be due to reduction of inhibition originating from inhibitory cell types other than PV and SOM or from multiple types of interneurons [40]. Our results showing intact miniature inhibition but deficient inhibition by Nkx2.1 cells using interneuron-selective optogenetic stimulation raise the possibility that deficit in PV or SOM inhibition may be present. Another group recently reported that conditional heterozygous *Tsc1* deletion in SOM interneurons resulted in neocortical layer 5 SOM interneuron mTORC1 hyperactivation, intact cell number, and soma size and increased PV co-expression in a subset of SOM cells [41]. However, spontaneous and miniature synaptic inhibition, as well as optogenetically evoked SOM interneuron inhibition of pyramidal cells was also unchanged in these mice [41]. In contrast, conditional homozygous *Tsc1* deletion in SOM interneurons resulted in impaired spontaneous, miniature, and optogenetically evoked SOM inhibition of pyramidal cells [41]. These results indicate that neocortical pyramidal cell inhibition generated by SOM interneurons is highly sensitive to *Tsc1* gene dosage in SOM cells. However, a major difference between these studies and ours is that with Nkx2.1-Cre driver mice *Tsc1* deletion occurs early in development, whereas with SOM- and PV-Cre mice, *Tsc1* deletion occurs post-natally. Thus, heterozygous *Tsc1* deletion early in development may be necessary for impairment in SOM and/or PV synaptic inhibition. This point may be relevant for TSC patients with germline or somatic mutations [5].

Finally, we observed correlated changes in hippocampal synaptic inhibition and cognitive impairments in mice with conditional *Tsc1* haploinsufficiency in Nkx2.1 cells. Thus, it is interesting to speculate that the behavioral changes may be due to the synaptic deficits. Indeed, impairment in hippocampal PV cell inhibition results in impaired spatial working memory but intact spatial learning and spatial reference memory [42]. Thus, the spatial working memory deficit we observed in mice with conditional *Tsc1* haploinsufficiency in Nkx2.1 cells is consistent with a deficit in PV cell inhibition. It is of interest that improvement, and not impairment, in spatial and contextual fear learning and memory were found in mice with conditional *Tsc1* haploinsufficiency in SOM interneurons [21], further suggesting that the behavioral changes observed in mice with conditional *Tsc1* haploinsufficiency in Nkx2.1 cells may predominantly originate from impairments in PV interneuron function. Further synaptic and behavioral studies with conditional *Tsc1* haploinsufficiency early in development and specifically in PV, SOM, and other interneurons will be important to dissect the specific contributions of different hippocampal interneuron types to the cellular and cognitive deficits in TSC mouse models.

## Limitations

A limitation of our work is that the experimental conditions with AAV-driven ChR2 expression used in the optogenetic experiments might lead to spurious results due to insufficient and/or variable expression of ChR2 across different experimental groups or even animals. New experiments could be performed to control for this potential source of variability and validate our findings by using mice with *Tsc1* haploinsufficiency expressing Cre-dependent ChR2 under the control of the Nkx2.1 promoter to ensure that the level of expression and overall population of ChR2 expressing cells would be the same across conditions/mice.

Another limitation of our study is the small sample size in some groups of the experiments with patch-clamp recordings of optogenetically evoked IPSCs. The small sample size in some groups is due to the variability of successful experiments in the different groups during interleaved experiments for which the experimenter was blind to genotype and treatment conditions. The differences in IPSC amplitude among groups suggest that there could be variability in ChR2 expression using our experimental design. Thus, additional experiments using the triple transgenic mice with stable ChR2 expression across the Nkx2.1 cell population with *Tsc1* haploinsufficiency would address also this limitation.

## Conclusions

We found that *Tsc1* haploinsufficiency in the MGE-derived Nkx2.1-expressing inhibitory cells upregulates mTORC1 activity in these interneurons in the hippocampus, reduces their synaptic inhibition of pyramidal cells, and alters contextual fear discrimination and spatial working memory. Thus, selective dysregulation of mTORC1 function in Nkx2.1 inhibitory cells appears sufficient to impair synaptic inhibition and contributes to cognitive deficits in the *Tsc1* mouse model of TSC. Specifically targeting mTORC1 function in inhibitory cells may thus represent an interesting novel strategy for addressing specific cognitive impairments due to TSC haploinsufficiency in ASD.

## Data Availability

The datasets used and/or analyzed during the current study are available from the corresponding author on reasonable request.

## Abbreviations

AAV: Adeno-associated virus
ACSF: Artificial cerebrospinal fluid
AHP: Afterhyperpolarization
AP: Action potential
ASD: Autism spectrum disorders
CGE: Caudal ganglionic eminence
EYFP: Enhanced yellow fluorescent protein
IPSC: Inhibitory postsynaptic current
LTD: Long-term depression
LTP: Long-term potentiation
mEPSC: Miniature excitatory postsynaptic current
MGE: Medial ganglionic eminence
mIPSC: Miniature inhibitory postsynaptic current
mTORC1: Mechanistic target of rapamycin complex 1
Nkx2.1: NK2 homeobox 1
nNOS: Neuronal nitric oxide synthase
PV: Parvalbumin
SOM: Somatostatin
Tg: Transgenic
TSC: Tuberous sclerosis complex
Vm: Resting membrane potential
Wt: Wild-type
